# 1-(4-Acetyl­phen­yl)-3-butyrylthio­urea

**DOI:** 10.1107/S1600536808022095

**Published:** 2008-07-23

**Authors:** Sohail Saeed, Moazzam Hussain Bhatti, Uzma Yunus, Peter G. Jones

**Affiliations:** a Department of Chemistry, Allama Iqbal Open University, Islamabad, Pakistan; b Institut für Anorganische und Analytische Chemie, Technische Universität Braunschweig, Postfach 3329, 38023 Braunschweig, Germany

## Abstract

The title compound, C_13_H_16_N_2_O_2_S, crystallizes in the thio­amide form with an intra­molecular hydrogen bond of type N—H⋯O_butyr­yl_. Mol­ecules are linked into chains parallel to [10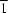] by a further hydrogen bond of type N—H⋯O_acet­yl_. C—H⋯O and C—H⋯S hydrogen bonds are also present.

## Related literature

For related literature, see: D’hooghe *et al.* (2005 ▶); Glasser & Doughty (1964 ▶); Huebner *et al.* (1953 ▶); Jain & Rao (2003 ▶); Morales *et al.* (2000 ▶); Ru *et al.* (1994 ▶); Xu *et al.* (2004 ▶); Xue *et al.* (2003 ▶); Zeng *et al.* (2003 ▶); Zheng *et al.* (2004 ▶); Douglas & Dains (1934 ▶).
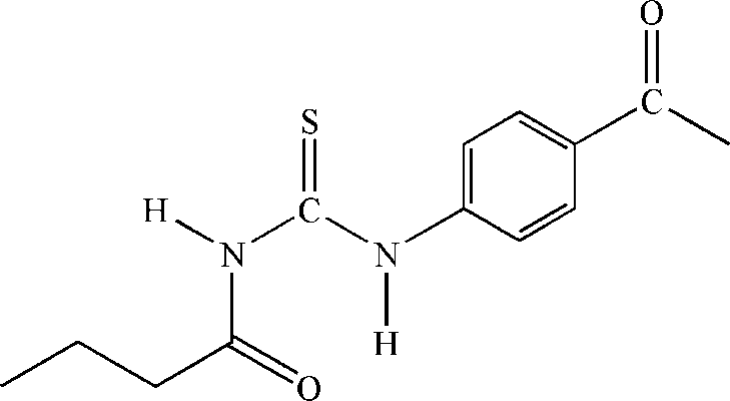


## Experimental

### 

#### Crystal data

C_13_H_16_N_2_O_2_S
*M* *_r_* = 264.34
Triclinic, 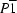

*a* = 7.5111 (5) Å
*b* = 9.7585 (8) Å
*c* = 10.5036 (5) Å
α = 65.283 (5)°
β = 76.245 (4)°
γ = 68.589 (5)°
*V* = 647.78 (8) Å^3^
*Z* = 2
Mo *K*α radiation
μ = 0.25 mm^−1^
*T* = 100 (2) K
0.35 × 0.20 × 0.10 mm

#### Data collection

Oxford Diffraction Xcalibur S diffractometer
Absorption correction: multi-scan (*CrysAlis RED*; Oxford Diffraction, 2008 ▶) *T*_min_ = 0.940, *T*_max_ = 0.976
22401 measured reflections
3613 independent reflections
3036 reflections with *I* > 2σ(*I*)
*R*_int_ = 0.030

#### Refinement

*R*[*F* ^2^ > 2σ(*F* ^2^)] = 0.031
*wR*(*F* ^2^) = 0.087
*S* = 1.06
3613 reflections
173 parameters
H atoms treated by a mixture of independent and constrained refinement
Δρ_max_ = 0.45 e Å^−3^
Δρ_min_ = −0.22 e Å^−3^

### 

Data collection: *CrysAlis CCD* (Oxford Diffraction, 2008 ▶); cell refinement: *CrysAlis RED* (Oxford Diffraction, 2008 ▶); data reduction: *CrysAlis RED*; program(s) used to solve structure: *SHELXS97* (Sheldrick, 2008 ▶); program(s) used to refine structure: *SHELXL97* (Sheldrick, 2008 ▶); molecular graphics: *XP* (Siemens, 1994 ▶); software used to prepare material for publication: *SHELXL97*.

## Supplementary Material

Crystal structure: contains datablocks I, global. DOI: 10.1107/S1600536808022095/pk2104sup1.cif

Structure factors: contains datablocks I. DOI: 10.1107/S1600536808022095/pk2104Isup2.hkl

Additional supplementary materials:  crystallographic information; 3D view; checkCIF report

## Figures and Tables

**Table 1:** Hydrogen-bond geometry (Å, °)

*D*—H⋯*A*	*D*—H	H⋯*A*	*D*⋯*A*	*D*—H⋯*A*
N2—H02⋯O1	0.840 (16)	1.874 (16)	2.6211 (12)	147.4 (16)
N1—H01⋯O2^i^	0.835 (16)	2.087 (16)	2.9057 (12)	166.7 (13)
C3—H3*B*⋯O2^i^	0.99	2.54	3.1345 (13)	118
C1—H1*C*⋯S^ii^	0.98	3.01	3.8996 (13)	151
C3—H3*A*⋯S^ii^	0.99	2.92	3.8444 (11)	155

Symmetry codes: (i) 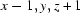; (ii) 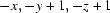.

**Fig. 1.**
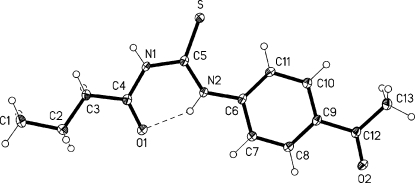
The molecule of the title compound in the crystal. Ellipsoids represent 50% probability levels.

**Fig. 2.**
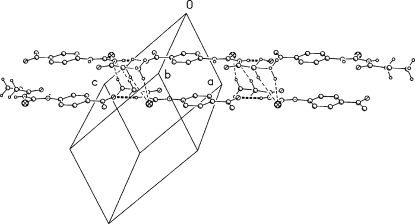
Packing diagram of the title compound showing classical and "weak" H bonds as thick and thin dashed bonds respectively. H atoms not involved in H bonds are omitted for clarity.

## supplementary crystallographic information

### Comment

Thiourea and its derivatives have found extensive applications in the fields of medicine, agriculture and analytical chemistry. Substituted thioureas are an important class of compounds, precursors or intermediates towards the synthesis of a variety of heterocyclic systems such as imidazole-2-thiones (Zeng *et al.*, 2003), 2-imino-1, 3-thiazolines (D'hooghe *et al.*, 2005) pyrimidines-2-thione (Jain & Rao, 2003) and (benzothiazolyl)-4-quinazolinones. N– (Substituted phenyl)-*N*-phenylthioureas and N– (substituted butanoyl)-*N*-phenylthioureas have been developed. Thioureas are also known to exhibit a wide range of biological activities including antiviral, antibacterial, antifungal, (Huebner *et al.*, 1953) antitubercular, antithyroidal, herbicidal and insecticidal activities and as agrochemicals (Xu *et al.*, 2004), *e.g.* 1-benzoyl-3-(4,5-disubstituted-pyrimidine-2-yl)- thioureas, which have excellent herbicidal activity (Zheng *et al.*, 2004). Thioureas are also well known chelating agents for transition metals (Xue *et al.*, 2003). *N*,*N*-Dialkyl-*N*'-benzoyl thioureas act as selective complexing agents for the enrichment of platinum metals even from strongly interfacing matrixes (Ru *et al.*, 1994). The complexes of thiourea derivatives also show various biological activities (Glasser & Doughty, 1964). Thioureas and substituted thioureas are also known as epoxy resin curing agents.

The title compound is a precursor for an attempt to synthesize imidazole derivatives and transition metal complexes as epoxy resin curing agents and accelerators. It crystallizes in the thioamide form (Fig. 1). The molecule is essentially planar (r.m.s. deviation of all non-H atoms 0.118 (1) Å), as reflected by the torsion angles O1—C4—N1—C5, C4—N1—C5—S and C4—N1—C5—N2 of 0.85 (17)°, 174.53 (8)° and -5.70 (15)°, respectively. The C4—O1, C5—S and C12—O2 bonds show a typical double bond character with bond lengths of 1.2246 (13), 1.6629 (11) and 1.2243 (13) Å, respectively. All the C—N bonds, C4—N1 = 1.3864 (13), C6—N2 = 1.4061 (12), C5—N2 = 1.3458 (13) and C5—N1 = 1.3948 (12) Å display a partial double bond character. Among the latter three C—N bonds, C4—N1 is the longest indicating a C(*sp*^2^)—N(*sp*^2^) single bond, while C5—N2 is the shortest bond with more double bond character. This demonstrates that there is π conjugation along S—C5—N2 but not along O1—C4—N1 and C4—N1—C5 as found in 1-(3-methoxybenzoyl)-3, 3-diethylthiourea (Morales *et al.*, 2000). There is a strong intramolecular hydrogen bond N2—H02···O1, with H2···O1 = 1.874 (16) Å, forming a 6-membered ring.

Molecules are connected in chains parallel to [101] by classical hydrogen bonds N1—H1···O2 and a weak bifurcated component C3—H3B···O2; the chains are further connected in an antiparallel sense by a bifurcated system of two C—H···S contacts (Table 2, Fig. 2).

### Experimental

The title compound was synthesized by a slight modification of the published procedure (Douglas & Dains, 1934). A solution of butanoyl chloride (0.1 mol) in dry acetone (75 ml) was added dropwise to a suspension of ammonium thiocyanate (0.1 mol) in dry acetone (55 ml) and the reaction mixture was refluxed for 45 minutes. After cooling to room temperature, a solution of 4-aminoacetophenone (0.1 mol) in dry acetone (25 ml) was added and the resulting mixture refluxed for 1.5 hrs. The reaction mixture was poured into five times its volume of cold water whereupon the thiourea precipitated as a solid. The product was recrystallized from ethyl acetate as colourless crystals (2.85 g, 79%). m.p.458 K.

### Refinement

H atoms of NH groups were refined freely. Methyl H atoms were included on the basis of idealized rigid groups (C—H 0.98 Å, H—C—H 109.5°) allowed to rotate but not tip. Other hydrogen atoms were included using a riding model with C—H 0.95 (aromatic) or 0.99 (methylene) Å. U(H) values were fixed at 1.5*U*_iso_(C) of the parent C atom for methyl H, 1.2*U*_iso_(C) for other H.

### Crystal data

**Table:**

C_13_H_16_N_2_O_2_S	*Z* = 2
*M**_r_* = 264.34	*F*_000_ = 280
Triclinic, *P*1	*D*_x_ = 1.355 Mg m^−^^3^
Hall symbol: -P 1	Melting point: 458 K
*a* = 7.5111 (5) Å	Mo *K*α radiation λ = 0.71073 Å
*b* = 9.7585 (8) Å	Cell parameters from 13985 reflections
*c* = 10.5036 (5) Å	θ = 2.6–30.6º
α = 65.283 (5)º	µ = 0.25 mm^−^^1^
β = 76.245 (4)º	*T* = 100 (2) K
γ = 68.589 (5)º	Tablet, pale yellow
*V* = 647.78 (8) Å^3^	0.35 × 0.20 × 0.10 mm

### Data collection

**Table:**

Oxford Diffraction Xcalibur S diffractometer	3613 independent reflections
Radiation source: Enhance (Mo) X-ray Source	3036 reflections with *I* > 2σ(*I*)
Monochromator: graphite	*R*_int_ = 0.030
Detector resolution: 16 pixels mm^-1^	θ_max_ = 30.7º
*T* = 100(2) K	θ_min_ = 2.6º
ω scans	*h* = −10→10
Absorption correction: multi-scan(CrysAlis RED; Oxford Diffraction, 2008)	*k* = −13→13
*T*_min_ = 0.940, *T*_max_ = 0.976	*l* = −15→15
22401 measured reflections	

### Refinement

**Table:**

Refinement on *F*^2^	Secondary atom site location: difference Fourier map
Least-squares matrix: full	Hydrogen site location: inferred from neighbouring sites
*R*[*F*^2^ > 2σ(*F*^2^)] = 0.031	H atoms treated by a mixture of independent and constrained refinement
*wR*(*F*^2^) = 0.087	*w* = 1/[σ^2^(*F*_o_^2^) + (0.0536*P*)^2^ + 0.0789*P*] where *P* = (*F*_o_^2^ + 2*F*_c_^2^)/3
*S* = 1.06	(Δ/σ)_max_ = 0.003
3613 reflections	Δρ_max_ = 0.45 e Å^−^^3^
173 parameters	Δρ_min_ = −0.22 e Å^−^^3^
Primary atom site location: structure-invariant direct methods	Extinction correction: none

### Special details

**Table:**

Geometry. All e.s.d.'s (except the e.s.d. in the dihedral angle between two l.s. planes) are estimated using the full covariance matrix. The cell e.s.d.'s are taken into account individually in the estimation of e.s.d.'s in distances, angles and torsion angles; correlations between e.s.d.'s in cell parameters are only used when they are defined by crystal symmetry. An approximate (isotropic) treatment of cell e.s.d.'s is used for estimating e.s.d.'s involving l.s. planes.
Refinement. Refinement of *F*^2^ against ALL reflections. The weighted *R*-factor *wR* and goodness of fit *S* are based on *F*^2^, conventional *R*-factors *R* are based on *F*, with *F* set to zero for negative *F*^2^. The threshold expression of *F*^2^ > σ(*F*^2^) is used only for calculating *R*-factors(gt) *etc*. and is not relevant to the choice of reflections for refinement. *R*-factors based on *F*^2^ are statistically about twice as large as those based on *F*, and *R*- factors based on ALL data will be even larger.

### Fractional atomic coordinates and isotropic or equivalent isotropic displacement parameters (Å^2^)

**Table:**

	*x*	*y*	*z*	*U*_iso_*/*U*_eq_	
S	0.29224 (4)	0.76793 (3)	0.30824 (3)	0.01606 (9)	
O1	0.44460 (12)	0.23350 (9)	0.43763 (8)	0.01882 (17)	
O2	1.01149 (11)	0.57384 (9)	−0.32905 (8)	0.01832 (17)	
N1	0.30196 (13)	0.47376 (10)	0.46308 (9)	0.01281 (18)	
H01	0.223 (2)	0.5166 (17)	0.5163 (15)	0.020 (3)*	
N2	0.50761 (13)	0.49957 (11)	0.26018 (9)	0.01395 (18)	
H02	0.522 (2)	0.4018 (19)	0.2950 (17)	0.032 (4)*	
C1	0.19337 (17)	0.00779 (13)	0.85964 (12)	0.0201 (2)	
H1A	0.2003	0.0630	0.9169	0.030*	
H1B	0.2504	−0.1064	0.9066	0.030*	
H1C	0.0587	0.0302	0.8482	0.030*	
C2	0.30349 (17)	0.06471 (12)	0.71538 (12)	0.0192 (2)	
H2A	0.4416	0.0343	0.7261	0.023*	
H2B	0.2907	0.0134	0.6556	0.023*	
C3	0.22642 (15)	0.24316 (12)	0.64468 (11)	0.0149 (2)	
H3A	0.0909	0.2712	0.6285	0.018*	
H3B	0.2281	0.2925	0.7096	0.018*	
C4	0.33675 (14)	0.31196 (12)	0.50663 (11)	0.0134 (2)	
C5	0.37498 (14)	0.57496 (12)	0.33954 (10)	0.01180 (19)	
C6	0.60770 (14)	0.55630 (12)	0.12737 (10)	0.01210 (19)	
C7	0.70848 (15)	0.44211 (12)	0.06708 (11)	0.0145 (2)	
H7	0.7064	0.3359	0.1171	0.017*	
C8	0.81080 (15)	0.48184 (12)	−0.06408 (11)	0.0145 (2)	
H8	0.8775	0.4033	−0.1039	0.017*	
C9	0.81661 (14)	0.63750 (12)	−0.13858 (11)	0.0126 (2)	
C10	0.71839 (15)	0.74982 (12)	−0.07705 (11)	0.0148 (2)	
H10	0.7227	0.8555	−0.1264	0.018*	
C11	0.61396 (15)	0.71148 (12)	0.05482 (11)	0.0148 (2)	
H11	0.5478	0.7900	0.0949	0.018*	
C12	0.92871 (14)	0.67675 (12)	−0.27915 (11)	0.0143 (2)	
C13	0.93843 (19)	0.84247 (14)	−0.35931 (12)	0.0241 (3)	
H13A	1.0270	0.8463	−0.4451	0.036*	
H13B	0.9845	0.8759	−0.3005	0.036*	
H13C	0.8102	0.9136	−0.3848	0.036*	

### Atomic displacement parameters (Å^2^)

**Table:**

	*U*^11^	*U*^22^	*U*^33^	*U*^12^	*U*^13^	*U*^23^
S	0.01598 (13)	0.01249 (13)	0.01624 (14)	−0.00382 (9)	0.00445 (9)	−0.00563 (10)
O1	0.0236 (4)	0.0161 (4)	0.0159 (4)	−0.0068 (3)	0.0050 (3)	−0.0081 (3)
O2	0.0209 (4)	0.0178 (4)	0.0143 (4)	−0.0047 (3)	0.0052 (3)	−0.0086 (3)
N1	0.0140 (4)	0.0132 (4)	0.0103 (4)	−0.0044 (3)	0.0036 (3)	−0.0058 (3)
N2	0.0168 (4)	0.0127 (4)	0.0110 (4)	−0.0054 (3)	0.0035 (3)	−0.0050 (3)
C1	0.0255 (5)	0.0160 (5)	0.0161 (5)	−0.0089 (4)	0.0005 (4)	−0.0023 (4)
C2	0.0232 (5)	0.0127 (5)	0.0181 (5)	−0.0055 (4)	0.0031 (4)	−0.0050 (4)
C3	0.0153 (5)	0.0142 (5)	0.0127 (5)	−0.0054 (4)	0.0024 (4)	−0.0037 (4)
C4	0.0137 (4)	0.0148 (5)	0.0120 (5)	−0.0052 (4)	−0.0007 (4)	−0.0048 (4)
C5	0.0112 (4)	0.0149 (5)	0.0100 (5)	−0.0049 (3)	−0.0002 (3)	−0.0048 (4)
C6	0.0117 (4)	0.0153 (5)	0.0095 (4)	−0.0050 (4)	0.0010 (3)	−0.0051 (4)
C7	0.0163 (5)	0.0132 (5)	0.0138 (5)	−0.0052 (4)	0.0017 (4)	−0.0059 (4)
C8	0.0148 (4)	0.0148 (5)	0.0139 (5)	−0.0041 (4)	0.0013 (4)	−0.0072 (4)
C9	0.0124 (4)	0.0155 (5)	0.0099 (5)	−0.0043 (4)	0.0001 (4)	−0.0051 (4)
C10	0.0171 (5)	0.0139 (5)	0.0126 (5)	−0.0054 (4)	0.0017 (4)	−0.0052 (4)
C11	0.0172 (5)	0.0141 (5)	0.0131 (5)	−0.0044 (4)	0.0024 (4)	−0.0073 (4)
C12	0.0143 (4)	0.0165 (5)	0.0112 (5)	−0.0048 (4)	0.0008 (4)	−0.0054 (4)
C13	0.0345 (6)	0.0192 (5)	0.0167 (5)	−0.0126 (5)	0.0104 (5)	−0.0077 (4)

### Geometric parameters (Å, °)

**Table:**

S—C5	1.6629 (11)	C12—C13	1.4993 (15)
O1—C4	1.2246 (13)	N1—H01	0.835 (16)
O2—C12	1.2243 (13)	N2—H02	0.840 (16)
N1—C4	1.3864 (13)	C1—H1A	0.9800
N1—C5	1.3948 (12)	C1—H1B	0.9800
N2—C5	1.3458 (13)	C1—H1C	0.9800
N2—C6	1.4061 (12)	C2—H2A	0.9900
C1—C2	1.5245 (15)	C2—H2B	0.9900
C2—C3	1.5185 (14)	C3—H3A	0.9900
C3—C4	1.5036 (14)	C3—H3B	0.9900
C6—C11	1.3949 (14)	C7—H7	0.9500
C6—C7	1.4008 (14)	C8—H8	0.9500
C7—C8	1.3807 (14)	C10—H10	0.9500
C8—C9	1.3999 (14)	C11—H11	0.9500
C9—C10	1.3923 (14)	C13—H13A	0.9800
C9—C12	1.4856 (14)	C13—H13B	0.9800
C10—C11	1.3921 (14)	C13—H13C	0.9800
			
C4—N1—C5	128.62 (9)	H1A—C1—H1B	109.5
C5—N2—C6	131.77 (9)	C2—C1—H1C	109.5
C3—C2—C1	110.45 (9)	H1A—C1—H1C	109.5
C4—C3—C2	114.32 (8)	H1B—C1—H1C	109.5
O1—C4—N1	122.98 (9)	C3—C2—H2A	109.6
O1—C4—C3	123.40 (9)	C1—C2—H2A	109.6
N1—C4—C3	113.60 (9)	C3—C2—H2B	109.6
N2—C5—N1	113.62 (9)	C1—C2—H2B	109.6
N2—C5—S	128.35 (8)	H2A—C2—H2B	108.1
N1—C5—S	118.03 (7)	C4—C3—H3A	108.7
C11—C6—C7	119.43 (9)	C2—C3—H3A	108.7
C11—C6—N2	125.91 (9)	C4—C3—H3B	108.7
C7—C6—N2	114.66 (9)	C2—C3—H3B	108.7
C8—C7—C6	120.86 (9)	H3A—C3—H3B	107.6
C7—C8—C9	120.25 (10)	C8—C7—H7	119.6
C10—C9—C8	118.54 (9)	C6—C7—H7	119.6
C10—C9—C12	122.35 (9)	C7—C8—H8	119.9
C8—C9—C12	119.11 (9)	C9—C8—H8	119.9
C11—C10—C9	121.78 (9)	C11—C10—H10	119.1
C10—C11—C6	119.12 (9)	C9—C10—H10	119.1
O2—C12—C9	119.80 (9)	C10—C11—H11	120.4
O2—C12—C13	120.49 (9)	C6—C11—H11	120.4
C9—C12—C13	119.71 (9)	C12—C13—H13A	109.5
C4—N1—H01	115.4 (10)	C12—C13—H13B	109.5
C5—N1—H01	115.9 (10)	H13A—C13—H13B	109.5
C5—N2—H02	111.5 (11)	C12—C13—H13C	109.5
C6—N2—H02	116.4 (11)	H13A—C13—H13C	109.5
C2—C1—H1A	109.5	H13B—C13—H13C	109.5
C2—C1—H1B	109.5		
			
C1—C2—C3—C4	175.26 (9)	C6—C7—C8—C9	0.55 (16)
C5—N1—C4—O1	0.85 (17)	C7—C8—C9—C10	0.39 (15)
C5—N1—C4—C3	−177.92 (9)	C7—C8—C9—C12	179.66 (9)
C2—C3—C4—O1	18.45 (15)	C8—C9—C10—C11	−0.70 (16)
C2—C3—C4—N1	−162.79 (9)	C12—C9—C10—C11	−179.95 (10)
C6—N2—C5—N1	176.36 (10)	C9—C10—C11—C6	0.07 (16)
C6—N2—C5—S	−3.91 (17)	C7—C6—C11—C10	0.87 (15)
C4—N1—C5—N2	−5.70 (15)	N2—C6—C11—C10	−179.38 (10)
C4—N1—C5—S	174.53 (8)	C10—C9—C12—O2	−179.50 (10)
C5—N2—C6—C11	11.68 (18)	C8—C9—C12—O2	1.26 (15)
C5—N2—C6—C7	−168.56 (11)	C10—C9—C12—C13	0.06 (16)
C11—C6—C7—C8	−1.19 (16)	C8—C9—C12—C13	−179.18 (10)
N2—C6—C7—C8	179.03 (9)		

### Hydrogen-bond geometry (Å, °)

**Table:**

*D*—H···*A*	*D*—H	H···*A*	*D*···*A*	*D*—H···*A*
N2—H02···O1	0.840 (16)	1.874 (16)	2.6211 (12)	147.4 (16)
N1—H01···O2^i^	0.835 (16)	2.087 (16)	2.9057 (12)	166.7 (13)
C3—H3B···O2^i^	0.99	2.54	3.1345 (13)	118
C1—H1C···S^ii^	0.98	3.01	3.8996 (13)	151
C3—H3A···S^ii^	0.99	2.92	3.8444 (11)	155

Symmetry codes: (i) *x*−1, *y*, *z*+1; (ii) −*x*, −*y*+1, −*z*+1.
